# Peripartum Androgen-Related Hormones and Anogenital Distance in Preeclampsia: A Cross-Sectional Case-Control Study

**DOI:** 10.3390/biomedicines14102155

**Published:** 2026-09-23

**Authors:** Ayşe Yavuz, Ömer Faruk Öz, Mediha Kübra Ceylan, Benan Kahraman Ersoy, Fatma Atmaca

**Affiliations:** 1 Department of Obstetrics and Gynecology, Istanbul Training and Research Hospital, University of Health Sciences, Istanbul 34098, Türkiye; 2 Department of Obstetrics and Gynecology, Faculty of Medicine, Akdeniz University, Antalya 07070, Türkiye

**Keywords:** preeclampsia, testosterone, free androgen index, anogenital distance, sex hormone-binding globulin, pregnancy, endocrine biomarkers

## Abstract

**Background/Objectives:** Preeclampsia is associated with placental, vascular, and endocrine alterations. We compared peripartum maternal androgen-related hormone measures and anogenital distance (AGD) between women with preeclampsia and normotensive controls and assessed whether observed differences persisted after clinical and sampling-related adjustment. **Methods:** In this prospectively recruited cross-sectional case-control study, 129 women were assessed for eligibility during delivery admission, and 87 women with singleton pregnancies were included: 41 with preeclampsia and 46 normotensive controls. Serum total testosterone, estradiol, androstenedione, dehydroepiandrosterone sulfate, and sex hormone-binding globulin were measured, and the free androgen index (FAI) was calculated. AGD was assessed as anus-to-clitoris distance (AGDac) and anus-to-fourchette distance (AGDaf). Adjusted linear models used heteroscedasticity-consistent robust standard errors; hormone outcomes were log-transformed. Sensitivity analyses excluded at-delivery samples. **Results:** Women with preeclampsia had higher total testosterone (3.22 vs. 2.31 nmol/L; *p* = 0.032), higher FAI (0.95% vs. 0.64%; *p* = 0.007), lower mean AGDac (10.41 vs. 11.35 cm; *p* = 0.032), and lower mean AGDaf (3.20 vs. 3.72 cm; *p* = 0.008). In adjusted models, preeclampsia remained associated with higher total testosterone (+78.3%), higher FAI (+99.9%), shorter AGDac (−1.56 cm), and shorter AGDaf (−0.60 cm). After excluding at-delivery samples (*n* = 69), only the AGDac association remained statistically significant (−1.39 cm; 95% confidence interval, −2.62 to −0.16; *p* = 0.026). **Conclusions:** Peripartum endocrine and AGD differences were observed in preeclampsia. After exclusion of at-delivery samples, AGDac remained statistically significant, whereas the endocrine associations and AGDaf did not. These cross-sectional findings do not establish causality or predictive utility and warrant longitudinal confirmation.

## 1. Introduction

Preeclampsia is a multisystem hypertensive disorder of pregnancy and remains an important contributor to maternal and perinatal morbidity [1,2,3,4]. The underlying biology is heterogeneous and includes abnormal placentation, placental stress, systemic endothelial dysfunction, and altered maternal vascular responses [5,6,7,8]. Contemporary diagnostic criteria center on new-onset hypertension after 20 weeks accompanied by proteinuria or, in the absence of proteinuria, specified maternal end-organ dysfunction [9], reflecting the evolution of diagnostic criteria over time [10]. Established clinical risk factors for preeclampsia include maternal demographic, obstetric, cardiometabolic, and pregnancy-related factors, but endocrine and anthropometric correlates remain incompletely characterized [11,12]. Endocrine pathways may intersect with these mechanisms because androgens can influence vascular tone, nitric oxide signaling, inflammation, and placental function [13].

Several observational studies have reported higher maternal androgen levels in preeclampsia or in women who later develop preeclampsia, but findings vary according to gestational age, disease severity, assay methodology, and timing of sampling [14,15,16]. Pregnancy alters placental steroid production and maternal testosterone concentrations [17,18], while estrogen physiology and sex hormone-binding globulin (SHBG) also change substantially across gestation [19,20]. These physiologic shifts complicate interpretation of single peripartum hormone measurements. Thus, increased testosterone or free androgen index (FAI) in established preeclampsia may represent disease-related endocrine physiology rather than a stable pre-pregnancy phenotype.

Anogenital distance (AGD) is the distance from the anus to anterior genital landmarks. It is sexually dimorphic from birth [21,22] and has been studied as an anthropometric marker related to prenatal androgen exposure [23,24]. Maternal polycystic ovary syndrome has also been associated with altered AGD in newborn daughters [25]. In adult women, longer AGD has been associated with polycystic ovary syndrome, higher testosterone concentrations, and greater ovarian follicular number [26,27,28]. If maternal AGD reflected a stable androgen-related phenotype predisposing to preeclampsia, longer AGD might be expected; conversely, a shorter or discordant AGD finding in established disease would argue against that interpretation and would raise concern for peripartum measurement effects or disease-related tissue changes.

We compared peripartum serum androgen-related measures and maternal AGD between women with preeclampsia and normotensive controls. We also evaluated whether observed differences persisted after adjustment for maternal age, body mass index (BMI), parity, gestational age at delivery, fetal sex, delivery mode, and blood-sampling timing and in sensitivity analyses addressing the imbalanced sampling-time structure. The comparison of circulating androgen-related measures with AGD allowed us to assess directional concordance between biochemical and anthropometric signals. AGD was treated as an anthropometric research measure collected during delivery admission, not as evidence of a pre-pregnancy hyperandrogenic state. Accordingly, the study was designed to estimate cross-sectional associations rather than causal or predictive effects.

## 2. Materials and Methods

### 2.1. Study Design and Participants

This was a prospectively recruited cross-sectional case-control study conducted during delivery admission at a tertiary obstetrics unit between November 2020 and March 2021. Women aged 18 years or older with singleton pregnancies were eligible. During recruitment, 129 women were assessed for eligibility; 42 were excluded, with no overlap among exclusion categories: chronic hypertension (*n* = 27), endocrine disorders affecting androgen concentrations (*n* = 7), multiple gestation (*n* = 5), and major fetal anomaly (*n* = 3), leaving 87 participants for analysis. Cases were women diagnosed with preeclampsia, and controls were normotensive women with blood pressure values < 140/90 mmHg throughout pregnancy and no hypertensive disorder of pregnancy. Chronic hypertension was defined as hypertension predating pregnancy or diagnosed before 20 weeks of gestation. Endocrine disorders affecting androgen concentrations included previously diagnosed polycystic ovary syndrome, gestational ovarian conditions, pre-existing or neoplastic ovarian pathologies, adrenal disorders, and pituitary or other neuroendocrine disorders. Major fetal anomaly was defined as a structural anomaly of prenatal origin with significant medical, social, or cosmetic consequences, or a chromosomal abnormality; minor anomalies and isolated sonographic soft markers were not included. Anomalies were coded using the International Classification of Diseases, 10th Revision (ICD-10; Q00–Q99), and prenatally suspected anomalies of uncertain severity were confirmed postnatally. Participant flow is shown in Figure 1. The study was approved by the Clinical Research Ethics Committee of the University of Health Sciences İstanbul Training and Research Hospital (Decision No. 2492; approval date: 21 August 2020). Written informed consent was obtained from all participants.

Study size was determined by the number of eligible consenting women recruited during the study period. No formal a priori sample size calculation was performed.

### 2.2. Definitions and Clinical Data

Preeclampsia was defined according to the American College of Obstetricians and Gynecologists Practice Bulletin No. 222 as new-onset hypertension after 20 weeks of gestation, with systolic blood pressure ≥ 140 mmHg and/or diastolic blood pressure ≥ 90 mmHg on two occasions at least 4 h apart in a previously normotensive woman, accompanied by proteinuria or, in the absence of proteinuria, maternal end-organ dysfunction [9]. Blood pressure measurements were obtained according to the institution’s standardized obstetric blood-pressure measurement protocol. Severe-range hypertension (systolic blood pressure ≥ 160 mmHg and/or diastolic blood pressure ≥ 110 mmHg) was confirmed over a shorter interval when clinically necessary to facilitate timely treatment. Preeclampsia with severe features was classified by severe-range blood pressure and/or thrombocytopenia (platelet count < 100,000/µL), renal insufficiency (serum creatinine > 1.1 mg/dL or doubling of the serum creatinine concentration in the absence of other renal disease), impaired liver function (liver transaminases > 2 times the upper limit of normal or severe persistent right-upper-quadrant/epigastric pain unresponsive to medication), pulmonary edema, new-onset headache unresponsive to medication and not accounted for by alternative diagnoses, or visual disturbances [9]. No participant had eclampsia. Maternal age, body mass index (BMI), gravidity, parity, gestational age at delivery, fetal sex, mode of delivery, blood pressure, and neonatal birth weight were abstracted from medical records.

### 2.3. Hormone Measurements

Maternal venous blood was collected during delivery admission and classified relative to delivery as pre-delivery (<0 h), at delivery (0 h), or postpartum (>0 to 24 h). Samples were centrifuged at 4000 rpm for 10 min at 4 °C, and serum was stored at −40 °C until analysis. Total testosterone, estradiol, dehydroepiandrosterone sulfate (DHEAS), and sex hormone-binding globulin (SHBG) were measured on an automated chemiluminescence platform (Siemens ADVIA Centaur XP; Siemens Healthineers, Erlangen, Germany). Androstenedione was measured using radioimmunoassay (Beckman Coulter Immunotech, Prague, Czech Republic). Total testosterone was analyzed in nmol/L. FAI was calculated as total testosterone (nmol/L)/SHBG (nmol/L) × 100 [29]. Mode of delivery was recorded because of its potential relevance to the peripartum endocrine milieu and was included as a covariate in adjusted models. Exact storage duration and lot-specific analytical sensitivity, measuring range, intra-assay and inter-assay coefficients of variation, and pregnancy-specific validation data were not available in the archived study records. Eight estradiol results (four in each group) were recorded exactly as 3000 pg/mL despite the presence of higher values elsewhere in the dataset; the available records did not permit determination of whether these represented exact measurements or reporting-limit values. Assay platforms and measurement units are summarized in Supplementary Table S1.

### 2.4. Anogenital Distance Measurement

Maternal AGD was measured during the same delivery admission in the lithotomy position with hips abducted and flexed approximately 45 degrees. Using previously described adult female anatomical landmarks [27,28], two distances were recorded with a disposable ruler: anus-to-clitoris distance (AGDac) and anus-to-fourchette distance (AGDaf). Each measurement was performed once by a trained examiner. The precise timing of AGD measurement relative to labor and delivery was not recorded. The manufacturer-specified resolution of the disposable ruler was unavailable; in the dataset, AGDac values were recorded in 1 cm increments and AGDaf values predominantly in 0.5 cm increments. These increments reflect recorded data granularity rather than a documented instrument-resolution specification. Repeat measurements and intra-observer or inter-observer reliability assessments were not performed.

### 2.5. Bias and Measurement Considerations

Potential sources of bias included delivery-admission sampling, the marked between-group imbalance in blood-sampling timing, differences in gestational age and delivery mode, and peripartum tissue changes that could affect AGD. Medication exposures with the potential to influence androgen concentrations were not systematically captured and therefore could not be incorporated as an exclusion criterion or covariate. These issues were addressed through protocolized measurement, exclusion of women with chronic hypertension or known endocrine disorders affecting androgen levels, covariate adjustment, and post hoc sensitivity analyses where possible.

### 2.6. Statistical Analysis

Continuous variables were summarized as median (interquartile range) because several hormone variables were skewed; mean ± standard deviation was additionally reported for AGD to aid interpretation when medians were similar. Categorical variables were summarized as n/N (%). Unadjusted group comparisons used the Mann–Whitney U test for continuous variables and chi-square or Fisher exact tests for categorical variables. Main adjusted analyses used linear regression models with heteroscedasticity-consistent type 3 (HC3) robust standard errors. Hormone outcomes were natural-log transformed and back-transformed to percent differences; AGD outcomes were modeled in centimeters. Models were adjusted for maternal age, BMI, parity, gestational age at delivery, fetal sex, delivery mode, and blood-sampling timing. Two post hoc sensitivity analyses assessed dependence on the sampling-time structure: the models were repeated (i) without the blood-sampling timing covariate and (ii) after excluding all at-delivery samples. Spearman correlations between hormone measures and AGD were secondary analyses. Exploratory unadjusted subgroup comparisons were also performed by fetal sex and blood-sampling timing. Because no single primary outcome was prespecified, no formal adjustment for multiple comparisons was applied; nominal *p* values should therefore be interpreted cautiously. Two-sided *p* values < 0.05 were considered statistically significant. Statistical analyses were performed using Python 3.13.5 (Python Software Foundation, Wilmington, DE, USA), SciPy 1.17.0, and statsmodels 0.14.6.

Because no formal a priori sample-size calculation was performed, we additionally characterized the sensitivity afforded by the available sample size using a simple two-sample framework. With 41 women with preeclampsia and 46 controls, a two-sided α of 0.05 and 80% power corresponded to an approximate minimum detectable standardized between-group difference of Cohen’s *d* = 0.61. This value represents an 80%-power benchmark rather than a threshold for statistical significance; smaller effects may still reach nominal statistical significance in a particular sample but would be detected with lower probability under repeated sampling. This descriptive calculation does not replace an a priori sample-size calculation.

Analyses used available data; no imputation was performed. All variables included in the main adjusted models were available for the included participants.

## 3. Results

### 3.1. Participant and Sampling Characteristics

Eighty-seven women were included: 46 normotensive controls and 41 women with preeclampsia. Among the preeclampsia cases, 21/41 (51.2%) had severe features and 20/41 (48.8%) had preeclampsia without severe features. Blood-sampling timing differed markedly between groups: pre-delivery samples were obtained from 11 controls and 11 preeclampsia cases, at-delivery samples from 1 control and 17 preeclampsia cases, and postpartum (>0 to 24 h) samples from 34 controls and 13 preeclampsia cases (Table 1). Among postpartum samples, blood was collected at a median of 11 h after delivery (interquartile range, 5.25–15 h; range, 1–21 h) in controls, whereas all 13 postpartum samples in the preeclampsia group were obtained 5 h after delivery.

Women with preeclampsia had higher BMI, delivered earlier, had higher blood pressure values, and had lower neonatal birth weight than controls. Cesarean delivery was also more frequent in the preeclampsia group. Fetal sex distribution did not differ significantly (Table 1).

### 3.2. Hormone and Anogenital Distance Measures

In unadjusted comparisons, median total testosterone was higher in preeclampsia than in controls (3.22 [2.34–4.75] vs. 2.31 [1.25–3.91] nmol/L; *p* = 0.032), and FAI was also higher (0.95 [0.66–1.61]% vs. 0.64 [0.33–0.96]%; *p* = 0.007). Androstenedione was lower in the preeclampsia group in unadjusted analysis (2.24 [1.19–3.14] vs. 3.21 [2.01–4.46] ng/mL; *p* = 0.020), whereas estradiol, DHEAS, and SHBG did not differ significantly (Table 2).

AGD distributions also differed between groups. Although median AGDac was 11.0 cm in both groups, mean ± standard deviation was 10.41 ± 1.48 cm in preeclampsia and 11.35 ± 1.91 cm in controls (Mann–Whitney *p* = 0.032). AGDaf was lower in preeclampsia (median 3.0 [3.0–4.0] cm; mean 3.20 ± 0.72 cm) than in controls (median 3.8 [3.0–4.0] cm; mean 3.72 ± 0.91 cm; *p* = 0.008) (Table 2).

Spearman correlations between hormone measures and AGD are reported in Supplementary Table S2; nominal associations varied by group and AGD measure. Fetal-sex-stratified and blood-sampling-timing-stratified unadjusted comparisons are provided in Supplementary Tables S3 and S4, respectively.

### 3.3. Adjusted Associations

After adjustment for maternal age, BMI, parity, gestational age at delivery, fetal sex, delivery mode, and blood-sampling timing, preeclampsia was associated with higher total testosterone (78.3% difference; 95% confidence interval [CI], 5.2 to 202.4; *p* = 0.032) and higher FAI (99.9%; 95% CI, 8.2 to 269.2; *p* = 0.027). The adjusted association with androstenedione was no longer evident (2.2%; 95% CI, −36.8 to 65.5; *p* = 0.929). Preeclampsia was also associated with shorter AGDac (−1.56 cm; 95% CI, −2.68 to −0.45; *p* = 0.006) and shorter AGDaf (−0.60 cm; 95% CI, −1.14 to −0.06; *p* = 0.030). Figure 2 summarizes the adjusted associations with selected endocrine and AGD outcomes. Complete adjusted estimates for all study outcomes are provided in Supplementary Table S5.

### 3.4. Sensitivity Analyses

Removing blood-sampling timing from the covariate set produced similar effect estimates: total testosterone +72.9% (95% CI, 1.5 to 194.5; *p* = 0.044), FAI +90.5% (95% CI, 3.5 to 250.5; *p* = 0.038), AGDac −1.51 cm (95% CI, −2.57 to −0.46; *p* = 0.005), and AGDaf −0.59 cm (95% CI, −1.08 to −0.11; *p* = 0.017). In the more restrictive analysis excluding all at-delivery observations (1 control and 17 preeclampsia cases; *n* = 69), the AGDac association persisted (−1.39 cm; 95% CI, −2.62 to −0.16; *p* = 0.026), whereas total testosterone (+61.2%; 95% CI, −10.0 to 188.8; *p* = 0.109), FAI (+79.7%; 95% CI, −10.1 to 259.5; *p* = 0.097), and AGDaf (−0.56 cm; 95% CI, −1.19 to 0.06; *p* = 0.075) were attenuated and no longer statistically significant. Full sensitivity-analysis results are provided in Supplementary Table S6.

## 4. Discussion

In this prospectively recruited cross-sectional case-control study, the main adjusted models showed higher peripartum total testosterone and FAI and shorter maternal AGD measures in women with preeclampsia. After exclusion of at-delivery samples, only AGDac remained statistically significant, whereas total testosterone, FAI, and AGDaf were attenuated beyond the nominal 0.05 significance threshold. The endocrine findings were therefore more dependent on the peripartum sampling structure than the AGDac association.

The direction of the endocrine findings is broadly consistent with prior reports linking higher maternal androgen levels with preeclampsia or with subsequent development of preeclampsia [14,15,16]. Our data were obtained close to delivery, when placental steroid production and maternal testosterone concentrations [17,18] and estrogen physiology and SHBG [19,20] may change substantially. The attenuation of total testosterone and FAI after exclusion of at-delivery samples reinforces this concern. Higher total testosterone and FAI in the main models should therefore be viewed as peripartum endocrine associations rather than evidence that androgen excess preceded disease onset. A recent review summarized evidence that elevated testosterone and androgen-receptor signaling may contribute to vascular and placental dysfunction in preeclampsia [30]. A recent prospective case-control study of pregnancies complicated by intrauterine growth restriction similarly found altered maternal SHBG and FAI profiles, with the FAI difference attenuating in an antepartum-only analysis, further illustrating the sensitivity of peripartum endocrine comparisons to sampling phase [31].

The AGD findings also require caution. Prior literature links longer female AGD with greater prenatal androgen exposure and adult hyperandrogenic reproductive phenotypes [23,24,26,27,28]. In contrast, women with preeclampsia in our study had lower AGD values, particularly AGDac, despite higher peripartum total testosterone in the main models. These opposite directions need not represent a biological contradiction because the measures reflect different time windows: maternal AGD has been studied as an anthropometric marker related to prenatal androgen exposure, whereas serum testosterone near delivery reflects the current peripartum endocrine milieu. Disease-related edema, gestational-age differences, tissue stretching, delivery-related changes, measurement variability, and residual confounding may also affect peripartum AGD. Absolute AGD values should not be compared directly across studies without accounting for differences in anatomical landmarks, positioning, measurement devices, and recording precision; heterogeneity in AGD methodology has been emphasized in the literature [32]. A single peripartum AGD measurement should therefore not be interpreted as evidence of a pre-existing androgenized phenotype or as a clinically actionable biomarker for preeclampsia risk stratification.

Strengths of this study include the simultaneous assessment of biochemical androgen-related markers and two AGD measures in the same clinically defined cohort and sensitivity analyses addressing the pronounced imbalance in blood-sampling timing. The use of log-transformed hormone outcomes and HC3 robust standard errors also reduced sensitivity to skewness and heteroscedasticity.

Several limitations should be considered. The cross-sectional delivery-admission design cannot establish temporal sequence or whether hormone differences preceded preeclampsia. Blood-sampling timing was strongly imbalanced between groups, particularly at delivery (1 control versus 17 preeclampsia cases), resulting in limited overlap and potential model extrapolation; excluding these observations attenuated the total testosterone, FAI, and AGDaf findings. Gestational age at delivery and delivery mode also differed substantially between groups and may lie partly on the disease pathway rather than act only as confounders. The modest sample size, single-center tertiary setting, and delivery-admission design limit generalizability to other obstetric populations and earlier pregnancy time points.

AGD was measured once, and its exact timing relative to labor and delivery was not recorded. No intra-observer or inter-observer reliability assessment was performed, ruler resolution was undocumented, and the available values were recorded at relatively coarse increments (1 cm for AGDac and predominantly 0.5 cm for AGDaf). Peripartum edema, tissue stretching, episiotomy, prior vaginal birth, and other delivery-related changes were also not fully captured.

Total testosterone was measured by chemiluminescence immunoassay rather than liquid chromatography-tandem mass spectrometry (LC-MS/MS), which may be less analytically specific at low concentrations. Detailed assay-performance characteristics and the exact duration of storage at −40 °C were unavailable in the archived study records. Eight estradiol results were recorded exactly as 3000 pg/mL without sufficient information to determine whether this represented an exact value or a reporting threshold.

Medication exposures potentially affecting androgen concentrations were not systematically captured. Although women with previously diagnosed polycystic ovary syndrome were excluded, no dedicated screening protocol was used to identify previously unrecognized polycystic ovary syndrome or clinical hyperandrogenism, so residual confounding from unrecognized hyperandrogenic conditions cannot be excluded. No formal multiplicity adjustment was applied across the eight outcome models; nominal *p* values should therefore be interpreted cautiously.

## 5. Conclusions

In the main adjusted analyses, preeclampsia was associated with higher peripartum total testosterone and FAI and with lower maternal AGD measures. After exclusion of at-delivery samples, AGDac remained statistically significant, whereas total testosterone, FAI, and AGDaf did not. The discordant biochemical and anthropometric findings warrant caution when interpreting peripartum measures as markers of a stable androgen-related phenotype. Larger longitudinal studies with standardized antepartum sampling, repeated high-resolution AGD measurements, detailed assay validation, and careful assessment of delivery-related perineal factors are needed.

## Figures and Tables

**Figure 1:**
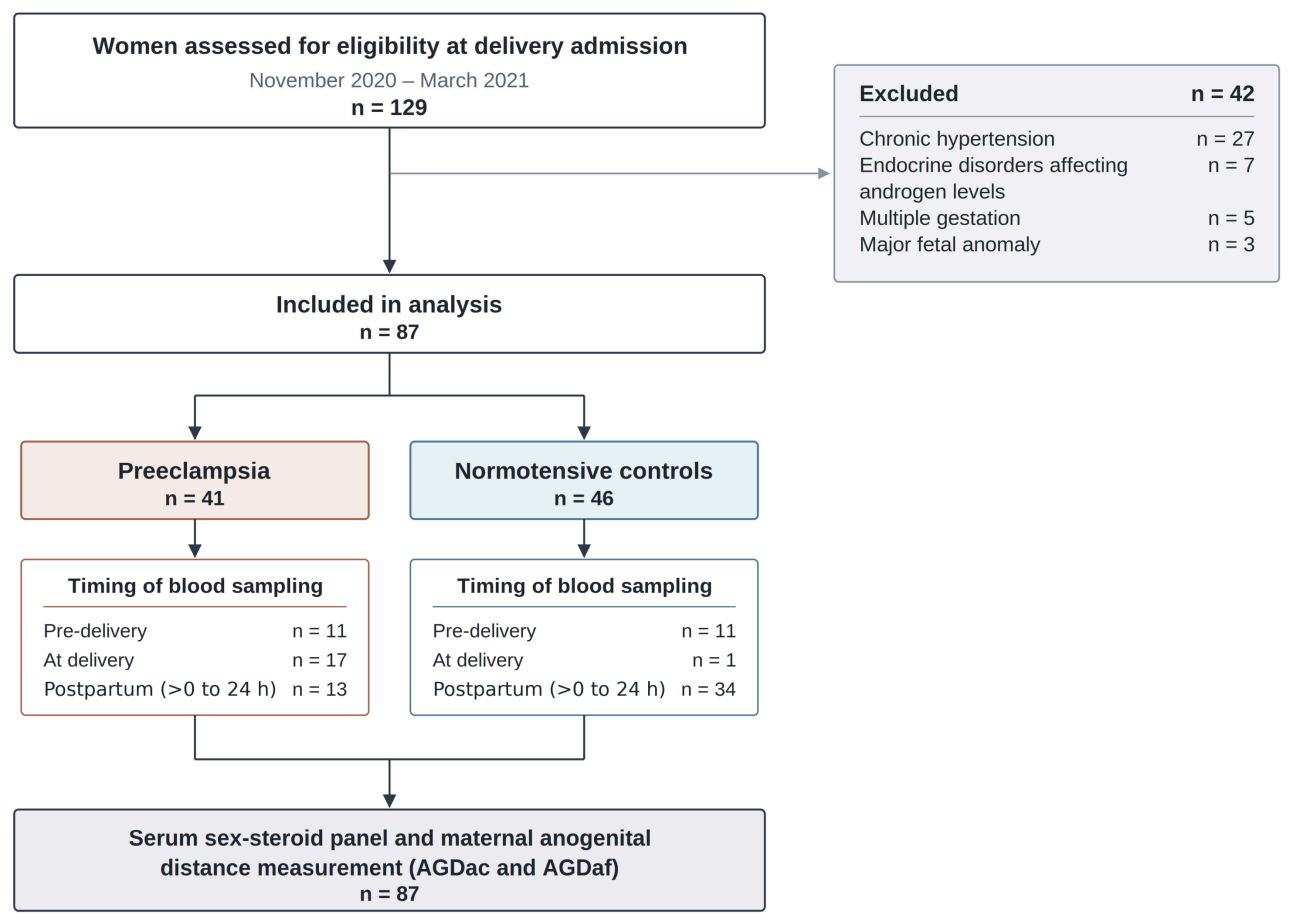
Participant flow, reasons for exclusion, study-group composition, and timing of blood sampling. Exclusion categories were mutually exclusive. AGDac, anus-to-clitoris distance; AGDaf, anus-to-fourchette distance.

**Figure 2:**
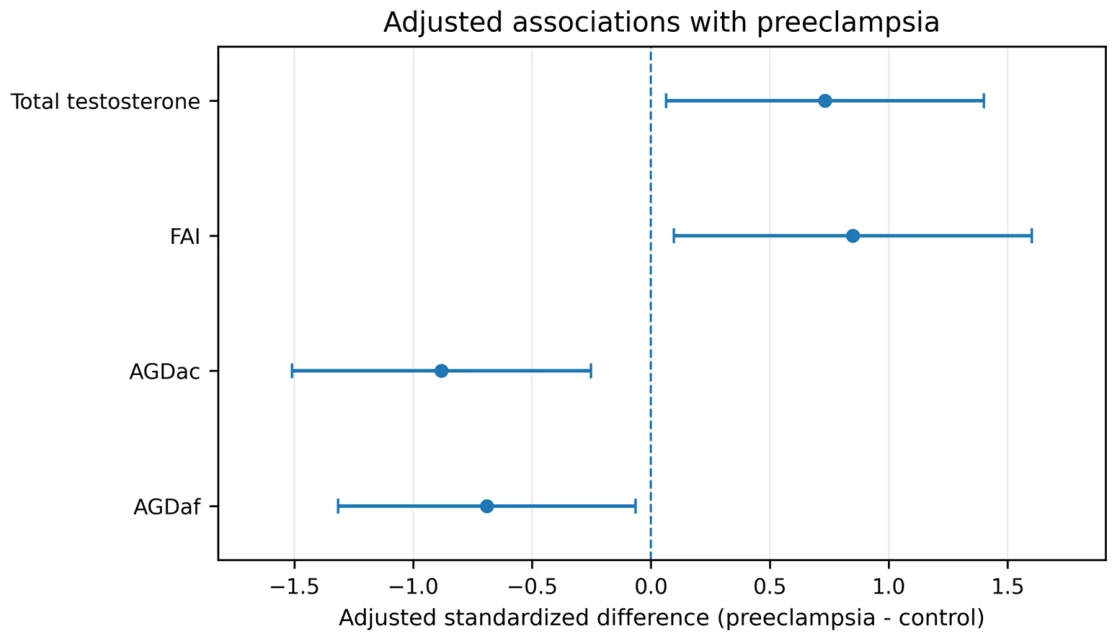
Adjusted standardized associations of preeclampsia with selected endocrine and anogenital distance outcomes. Points indicate adjusted standardized effect estimates for preeclampsia versus normotensive controls, and horizontal bars indicate 95% confidence intervals. Negative values indicate lower values in preeclampsia and higher values in controls, whereas positive values indicate higher values in preeclampsia. Models were adjusted for maternal age, body mass index, parity, gestational age at delivery, fetal sex, delivery mode, and blood-sampling timing. Total testosterone and FAI were natural-log transformed before standardization. AGDac, anus-to-clitoris distance; AGDaf, anus-to-fourchette distance; FAI, free androgen index.

**Table 1:** Maternal, perinatal, and sampling characteristics.

Characteristic	Control (*n* = 46)	Preeclampsia (*n* = 41)	*p* Value
Maternal age (years)	26.0 (23.0–33.0)	29.0 (26.0–37.0)	0.015
BMI (kg/m^2^)	28.5 (26.0–30.0)	31.0 (28.0–34.0)	0.003
Gravidity	3 (2–4)	2 (2–3)	0.101
Parity	2 (1–2)	1 (0–2)	0.028
Gestational age at delivery (weeks)	38.6 (37.9–39.4)	35.6 (32.9–37.0)	<0.001
Systolic blood pressure (mmHg)	110 (106–119)	170 (158–180)	<0.001
Diastolic blood pressure (mmHg)	72 (65–76)	100 (92–110)	<0.001
Birth weight (g)	3260 (2996–3468)	2420 (1900–2980)	<0.001
Sampling time from delivery (h)	6 (0–15)	0 (−3–5)	<0.001
Male fetus, n/N (%)	23/46 (50.0)	23/41 (56.1)	0.724
Cesarean delivery, n/N (%)	24/46 (52.2)	37/41 (90.2)	<0.001
Preeclampsia with severe features, n/N (%)	—	21/41 (51.2)	—
Blood-sampling timing, n (%)			<0.001
Pre-delivery	11 (23.9)	11 (26.8)	
At delivery	1 (2.2)	17 (41.5)	
Postpartum (>0 to 24 h)	34 (73.9)	13 (31.7)	

Values are median (interquartile range) unless otherwise specified. Categorical variables are n/N (%) or n (%). The *p* values were obtained with Mann–Whitney U tests for continuous variables and chi-square or Fisher exact tests for categorical variables. BMI, body mass index.

**Table 2:** Hormone measures, anogenital distance measures, and adjusted associations with preeclampsia.

Measure	Control (*n* = 46), Median (IQR); AGD Mean ± SD	Preeclampsia (*n* = 41), Median (IQR); AGD Mean ± SD	Unadjusted *p*	Adjusted PE Effect (95% CI)	Adjusted *p*
Total testosterone (nmol/L)	2.31 (1.25–3.91)	3.22 (2.34–4.75)	0.032	78.3% (5.2 to 202.4)	0.032
Estradiol (pg/mL)	885 (208–3000)	605 (153–3000)	0.356	−40.4% (−81.7 to 93.9)	0.390
Androstenedione (ng/mL)	3.21 (2.01–4.46)	2.24 (1.19–3.14)	0.020	2.2% (−36.8 to 65.5)	0.929
DHEAS (µg/dL)	102 (72–166)	111 (31–161)	0.133	−7.0% (−41.6 to 48.2)	0.760
SHBG (nmol/L)	368 (298–458)	344 (200–440)	0.201	−10.8% (−30.4 to 14.3)	0.367
FAI (%)	0.64 (0.33–0.96)	0.95 (0.66–1.61)	0.007	99.9% (8.2 to 269.2)	0.027
AGDac (cm)	11.0 (10.0–12.0); 11.35 ± 1.91	11.0 (9.0–12.0); 10.41 ± 1.48	0.032	−1.56 (−2.68 to −0.45)	0.006
AGDaf (cm)	3.8 (3.0–4.0); 3.72 ± 0.91	3.0 (3.0–4.0); 3.20 ± 0.72	0.008	−0.60 (−1.14 to −0.06)	0.030

Hormone summaries are median (interquartile range). AGD summaries are median (interquartile range); mean ± standard deviation. Adjusted effect represents preeclampsia vs. control. Hormone outcomes were natural-log transformed and back-transformed to percent differences. AGD outcomes were modeled in centimeters. Models adjusted for maternal age, BMI, parity, gestational age at delivery, fetal sex, delivery mode, and blood-sampling timing, using HC3 robust standard errors. AGD, anogenital distance; AGDac, anus-to-clitoris distance; AGDaf, anus-to-fourchette distance; BMI, body mass index; CI, confidence interval; DHEAS, dehydroepiandrosterone sulfate; FAI, free androgen index; HC3, heteroscedasticity-consistent type 3; IQR, interquartile range; PE, preeclampsia; SHBG, sex hormone-binding globulin. Routine laboratory reference intervals, provided for analytical context only and not used for participant classification, were: total testosterone 0.084–0.481 µg/L (equivalent to approximately 0.29–1.67 nmol/L), androstenedione 0.303–3.32 ng/mL, DHEAS 92.8–340 µg/dL, and SHBG 30–135 nmol/L. For estradiol, the laboratory used age- and menstrual-phase-dependent nonpregnant reference intervals; in adult nonpregnant women, the listed follicular, ovulatory, and luteal intervals collectively spanned 22.4–517 pg/mL. Pregnancy-specific reference intervals were not available. No routine laboratory reference interval was available for FAI, which was calculated from total testosterone and SHBG; a pregnancy-specific FAI reference interval was also unavailable. Because these intervals were derived from nonpregnant adults, they should not be used to classify values obtained during pregnancy as normal or abnormal.

## Data Availability

The data presented in this study are available on request from the corresponding author due to privacy and ethical restrictions.

## Supplementary Materials

The following supporting information can be downloaded at: https://www.mdpi.com/article/10.3390/biomedicines14102155/s1, Table S1: Assay platforms and measurement units; Table S2: Exploratory Spearman correlations between hormone measures and anogenital distance; Table S3: Fetal-sex-stratified unadjusted comparisons for selected outcomes; Table S4: Blood-sampling-timing-stratified unadjusted comparisons for selected outcomes; Table S5: Adjusted associations between preeclampsia and study outcomes; Table S6: Sensitivity analyses for blood-sampling timing.
