# Supplementary material for: Peripartum Androgen-Related Hormones and Anogenital Distance in Preeclampsia: A Cross-Sectional Case-Control Study
Source: Biomedicines. 2026 Sep 23;14(10):2155. doi: 10.3390/biomedicines14102155 (PMC13701652; doi:10.3390/biomedicines14102155)
Supplement: Supplementary file 1 [file biomedicines-14-02155-s001.zip › biomedicines-4563199-supplementary.pdf]

Supplementary Materials

Supplementary Table S1. Assay platforms and measurement units.

Marker	Platform/kit	Unit
Total testosterone	Siemens ADVIA Centaur XP; Siemens Healthineers	nmol/L
Estradiol	Siemens ADVIA Centaur XP; Siemens Healthineers	pg/mL
DHEAS	Siemens ADVIA Centaur XP; Siemens Healthineers	µg/dL
SHBG	Siemens ADVIA Centaur XP; Siemens Healthineers	nmol/L
Androstenedione	Beckman Coulter Immunotech RIA; Beckman Coulter	ng/mL

Hormones were measured using routine clinical laboratory platforms. Lot-specific analytical sensitivity, measuring range, intra-assay/inter-assay coefficients of variation, and pregnancy-specific validation information were not available in the archived study records; this limitation is described in the main manuscript. Routine laboratory reference intervals (not pregnancy-specific and not used for participant classification) were total testosterone 0.084-0.481 µg/L (approximately 0.29-1.67 nmol/L), androstenedione 0.303-3.32 ng/mL, DHEAS 92.8-340 µg/dL, and SHBG 30-135 nmol/L. For estradiol, the laboratory used age- and menstrual-phase-dependent nonpregnant reference intervals; for adult nonpregnant women the listed follicular, ovulatory, and luteal intervals collectively spanned 22.4-517 pg/mL. Pregnancy-specific reference intervals were not available. No routine laboratory reference interval was available for FAI, which was calculated from total testosterone and SHBG; a pregnancy-specific FAI reference interval was also unavailable. Because these intervals were derived from nonpregnant adults, they should not be used to classify values obtained during pregnancy as normal or abnormal.

Supplementary Table S2. Exploratory Spearman correlations between hormone measures and anogenital distance.

Hormone	Control AGDac rho (p)	Control AGDaf rho (p)	PE AGDac rho (p)	PE AGDaf rho (p)
Total testosterone	0.19 (0.218)	0.34 (0.022)	−0.07 (0.674)	0.18 (0.270)
Estradiol	0.04 (0.794)	0.28 (0.058)	0.16 (0.317)	−0.04 (0.815)
Androstenedione	0.08 (0.584)	0.41 (0.004)	0.34 (0.029)	0.32 (0.045)
DHEAS	0.02 (0.885)	0.05 (0.717)	−0.12 (0.473)	0.18 (0.271)
SHBG	0.08 (0.612)	0.31 (0.039)	0.15 (0.362)	−0.25 (0.120)
FAI	0.14 (0.357)	0.23 (0.131)	−0.12 (0.465)	0.28 (0.075)

Values are Spearman correlation coefficients with p values in parentheses. These analyses are exploratory and should not be interpreted causally. AGD, anogenital distance; AGDac, anus-to-clitoris distance; AGDaf, anus-to-fourchette distance; PE, preeclampsia.

Supplementary Table S3. Fetal-sex-stratified unadjusted comparisons for selected outcomes.

Fetal sex	Measure	Control median (IQR)	Preeclampsia median (IQR)	p value
Female	Total testosterone (nmol/L)	1.72 (1.26-3.31)	3.10 (2.34-4.15)	0.016
Female	FAI (%)	0.59 (0.36-0.90)	0.94 (0.64-1.52)	0.060
Female	AGDac (cm)	11.0 (10.0-12.5)	11.0 (9.2-12.0)	0.292
Female	AGDaf (cm)	3.5 (3.0-4.0)	3.0 (3.0-3.8)	0.068
Male	Total testosterone (nmol/L)	3.04 (1.31-4.65)	3.22 (2.04-5.15)	0.356
Male	FAI (%)	0.82 (0.30-1.10)	0.96 (0.68-1.71)	0.068

Fetal sex	Measure	Control median (IQR)	Preeclampsia median (IQR)	p value
Male	AGDac (cm)	12.0 (10.0-12.0)	11.0 (9.5-11.0)	0.059
Male	AGDaf (cm)	4.0 (3.0-4.0)	3.0 (3.0-4.0)	0.052

p values were obtained with Mann-Whitney U tests. These subgroup analyses are exploratory and underpowered.

Supplementary Table S4. Blood-sampling-timing-stratified unadjusted comparisons for selected outcomes.

Sampling timing	Measure	Control n; median (IQR)	Preeclampsia n; median (IQR)	p value
Pre-delivery	Total testosterone (nmol/L)	11; 2.31 (1.12-3.50)	11; 2.88 (1.72-3.45)	0.511
Pre-delivery	FAI (%)	11; 0.64 (0.32-0.85)	11; 0.69 (0.48-1.17)	0.555
Pre-delivery	AGDac (cm)	11; 11.0 (10.5-12.0)	11; 11.0 (10.5-12.0)	0.657
Pre-delivery	AGDaf (cm)	11; 3.0 (3.0-4.0)	11; 3.0 (3.0-4.0)	0.530
At delivery	Total testosterone (nmol/L)	1; 0.84	17; 3.22 (2.49-4.75)	NA
At delivery	FAI (%)	1; 0.20	17; 0.96 (0.79-1.66)	NA
At delivery	AGDac (cm)	1; 14.0	17; 11.0 (9.0-11.0)	NA
At delivery	AGDaf (cm)	1; 3.5	17; 3.0 (3.0-4.0)	NA
Postpartum (>0 to 24 h)	Total testosterone (nmol/L)	34; 2.32 (1.31-4.57)	13; 4.02 (3.06-8.74)	0.059
Postpartum (>0 to 24 h)	FAI (%)	34; 0.68 (0.34-1.11)	13; 1.21 (0.67-2.22)	0.017
Postpartum (>0 to 24 h)	AGDac (cm)	34; 11.5 (10.0-12.0)	13; 10.0 (9.0-11.0)	0.066
Postpartum (>0 to 24 h)	AGDaf (cm)	34; 4.0 (3.0-4.0)	13; 3.0 (3.0-4.0)	0.027

p values were obtained with Mann-Whitney U tests where both groups had sufficient observations. At-delivery between-group p values were not reported because only one control had an at-delivery sample.

Supplementary Table S5. Adjusted associations between preeclampsia and study outcomes.

Outcome	Scale	Adjusted effect	95% CI lower	95% CI upper	p value
Total testosterone	% difference	78.34	5.17	202.42	0.032
Estradiol	% difference	-40.40	-81.68	93.94	0.390
Androstenedione	% difference	2.23	-36.84	65.45	0.929
DHEAS	% difference	-7.00	-41.65	48.23	0.760
SHBG	% difference	-10.78	-30.35	14.30	0.367
FAI	% difference	99.89	8.22	269.19	0.027
AGDac	Mean difference	-1.56	-2.68	-0.45	0.006
AGDaf	Mean difference	-0.60	-1.14	-0.06	0.030

Models adjusted for maternal age, BMI, parity, gestational age at delivery, fetal sex, delivery mode, and blood-sampling timing with HC3 robust standard errors. Hormone outcomes were natural-log transformed and back-transformed to percent differences; AGD outcomes are in centimeters.

Supplementary Table S6. Sensitivity analyses for blood-sampling timing.

Outcome	Scale	Model without timing covariate: effect (95% CI); p	Excluding at-delivery samples (n=69): effect (95% CI); p	Interpretation
Total testosterone	% difference	72.9% (1.5 to 194.5); 0.044	61.2% (-10.0 to 188.8); 0.109	Attenuated after exclusion
Estradiol	% difference	-26.6% (-74.3 to 109.6); 0.564	-9.5% (-73.9 to 214.6); 0.876	Not statistically significant
Androstenedione	% difference	1.2% (-34.9 to 57.4); 0.957	-7.2% (-44.9 to 56.3); 0.778	Not statistically significant
DHEAS	% difference	-12.5% (-43.5 to 35.4); 0.548	4.6% (-33.1 to 63.6); 0.844	Not statistically significant
SHBG	% difference	-9.2% (-27.8 to 14.2); 0.410	-10.3% (-32.1 to 18.3); 0.441	Not statistically significant
FAI	% difference	90.5% (3.5 to 250.5); 0.038	79.7% (-10.1 to 259.5); 0.097	Attenuated after exclusion
AGDac	Mean difference, cm	-1.51 (-2.57 to -0.46); 0.005	-1.39 (-2.62 to -0.16); 0.026	Association persisted
AGDaf	Mean difference, cm	-0.59 (-1.08 to -0.11); 0.017	-0.56 (-1.19 to 0.06); 0.075	Attenuated after exclusion

Sensitivity models used the same covariates as the main adjusted analysis except as specified. The model without the timing covariate omitted blood-sampling timing. The exclusion analysis removed all 18 at-delivery observations (1 control and 17 preeclampsia cases) and retained blood-sampling timing as a two-level covariate (pre-delivery vs. postpartum (>0 to 24 h)). HC3 robust standard errors were used. CI, confidence interval; DHEAS, dehydroepiandrosterone sulfate; FAI, free androgen index; SHBG, sex hormone-binding globulin.
